# Presbyopia Correction in Lens Replacement Surgery: A Review

**DOI:** 10.1111/ceo.14535

**Published:** 2025-04-28

**Authors:** Benjamin Stern, Damien Gatinel

**Affiliations:** ^1^ Anterior Segment and Refractive Surgery Department Rothschild Foundation Hospital Paris France; ^2^ Department of Ophthalmology Hadassah‐Hebrew University Medical Center Jerusalem Israel

**Keywords:** cataract, extended depth‐of‐focus lenses, monovision, multifocal intraocular lenses, presbyopia

## Abstract

Presbyopia affects approximately 1.8 billion individuals globally, posing significant challenges as life expectancy and near‐vision demands, particularly with mobile phone use, grow. Addressing presbyopia during lens replacement surgery has become a key focus for cataract surgeons, aiming to reduce dependence on corrective eyewear. This review provides an overview of current intraocular lens (IOL) technologies and surgical strategies for presbyopia correction. Personalised decision‐making is essential, considering each patient's visual needs, expectations, and ocular anatomy. Partial correction approaches, such as monovision and extended depth‐of‐focus IOLs, can improve intermediate vision but involve specific trade‐offs compared to monofocal lenses, depending on the technology utilised. For complete presbyopia correction, multifocal IOLs remain the most effective option. A mix‐and‐match strategy involving unilateral multifocal implantation shows promise, while sulcus‐fixated supplementary IOLs offer the advantage of easier reversibility. Careful IOL selection is particularly important for patients with atypical ocular anatomy or coexisting conditions, which may be progressive.

## 1 Introduction

Lens replacement surgery, specifically involving the use of intraocular lenses (IOLs) to replace the crystalline lens, has markedly improved in both safety and precision in recent years. The rate of serious complications has decreased to approximately 0.5% [1], while refractive outcomes have shown significant advancement. Currently, 85% of patients achieve refractive error within 0.5 diopters of the target [2], compared to 60% in the early 2000s [3]. Some surgeons are even achieving success rates exceeding 92% [4].

Continuous advancements in IOL technology, marked by the annual introduction of new models, have extended the scope of cataract surgery beyond simple single‐focus correction to encompass effective presbyopia management—addressing the age‐related decline in accommodative function. These innovations have significantly transformed both patient expectations and surgical goals, as many patients now seek comprehensive visual rehabilitation. This rehabilitation aims not only to restore vision following cataract surgery but also to correct refractive errors and presbyopia, ultimately reducing or even eliminating dependence on glasses.

Enhanced safety and improved outcomes in cataract surgery have contributed significantly to the growing popularity of refractive lens exchange (RLE). This procedure, which aims to correct ametropia and presbyopia in patients without cataracts, has gained acceptance despite persistent concerns regarding the risk of retinal detachment, particularly in relatively younger patients. RLE offers the unique advantage of proactively preventing cataract formation while directly addressing the root cause of presbyopia—the crystalline lens, which undergoes age‐related stiffening and reduced flexibility [5, 6], leading to compromised near vision. Although corrective options such as glasses, contact lenses and corneal surgeries serve many patients effectively, RLE provides a durable and comprehensive solution for those seeking permanent vision correction.

This literature review, conducted as a non‐systematic review, involved searching PubMed for general articles using the keywords ‘(cataract OR intraocular lens) AND (presbyopia OR presbyopic)’, followed by examining references from the retrieved articles and performing subject‐specific searches in PubMed. It explores clinical strategies for presbyopia correction in lens replacement surgery and provides an in‐depth analysis of key clinical considerations, available options and emerging perspectives in this rapidly evolving field.

## 2 The Burden of Presbyopia and Its Treatment

Presbyopia affects approximately 1.8 billion people worldwide, impacting nearly one in four individuals [7] and significantly diminishing quality of life [8]. Typically manifesting in individuals in their mid‐40s, presbyopia has historically been associated with old age, as implied by its name, derived from the late 18th‐century Greek words presbys, meaning ‘old man’, and ops, meaning ‘to see like’ [9]. However, with evolving societal perceptions and increasing life expectancy, individuals in their 40s are now considered middle‐aged rather than elderly.

Life expectancy continues to rise, with projections for European countries indicating an increase from 78.3 years for men and 83.4 years for women in 2014 to 90.5 years for men and 92.8 years for women by 2065 [10]. Population aging is observed not only in Europe but also in developing countries such as India, where the proportion of individuals over 60 years old is projected to increase from 10.5% to 20.8% of the total population [11]. Consequently, the number of presbyopic patients is increasing globally, and the duration of time individuals live with presbyopia is also lengthening, as older adults remain active and employed for longer periods.

Moreover, older adults today engage in more frequent near‐vision tasks due to the widespread use of smartphones, which have become integral to modern life. Currently, individuals over the age of 60 spend an average of 2.4 h per day on smartphones [12], a figure expected to rise as the average American adult now spends 4.5 h daily on these devices [13], with older generations increasingly adopting similar usage patterns. Similar trends are observed globally, although the extent of smartphone usage may vary based on regional socioeconomic factors.

These demographic and technological changes underscore the importance of effective presbyopia management and correction to maintain independence and manage everyday tasks efficiently.

Conventional treatments for presbyopia typically involve the use of reading glasses or multifocal spectacles, such as bifocal or progressive lenses. While these options are generally considered safe and convenient, it is essential to evaluate their long‐term implications. For instance, multifocal spectacles have been associated with an increased risk of falls, posing a significant concern for older adults [14, 15, 16, 17]. In addition, reading glasses may present challenges for elderly individuals, particularly those with cognitive impairments, who may struggle to manage and use them consistently.

Corneal treatment options, such as contact lenses and refractive surgeries like presbyLASIK, may not be suitable for all patients. Dry eye syndrome, prevalent among older populations and frequently associated with blepharitis, can make contact lens use uncomfortable. Moreover, older adults may experience difficulties with the manual dexterity required for handling lenses. Corneal refractive surgery may also be inappropriate for some patients, as it can exacerbate dryness and only provide partial correction of presbyopia.

Nevertheless, multifocal contact lenses can be a good option for regular contact lens wearers, with 44% opting for them [18]. This adoption rate is significantly higher than that of presbyopia‐correcting IOLs implanted during cataract surgery, indicating substantial potential for increased utilisation of presbyopia‐correcting IOLs in the future. The relatively low uptake of these lenses appears to be due to limited patient awareness of surgical options and some surgeons' reluctance to allocate the additional time [19] and fees for these presbyopia‐correcting IOLs—barriers expected to diminish over time. It is important to note that multifocal technologies differ in design: IOLs use mainly diffractive optics, whereas multifocal contact lenses employ refractive optics. Age and ocular conditions are also significant factors, as older cataract patients may perceive presbyopia correction differently and often present with ocular pathologies that may contraindicate presbyopia‐correcting IOLs implantation.

Lens replacement surgery using presbyopia‐correcting IOLs offers a permanent solution for presbyopia, with the potential for full correction. In patients without cataracts, RLE not only addresses presbyopia but also eliminates the future risk of cataract formation. However, RLE remains an invasive procedure with an associated risk of retinal detachment, particularly in myopic patients. Phakic IOLs, a less invasive alternative, have shown promising outcomes [20] and may see greater adoption in the future.

For cataract patients, cataract removal minimises the perceived trade‐offs of light‐splitting technology in non‐accommodative presbyopia‐correcting IOLs, and patient satisfaction increases with higher cataract severity scores [21]. In contrast, patients undergoing RLE procedures, particularly younger individuals with very clear lenses, may experience reduced contrast sensitivity with multifocal IOLs [22]. Cataract surgery presents an interesting opportunity for presbyopia correction and is expected to become the standard of care, especially for patients with otherwise healthy eyes.

## 3 Strategies for Presbyopia Correction in Lens Replacement Surgery

A successful strategy for presbyopia correction should be a collaborative decision between the surgeon and the patient. The surgeon must provide an objective overview of all available options, delivering the most up‐to‐date and reliable information while remaining uninfluenced by manufacturers' marketing messages. To ensure patient satisfaction and optimal outcomes in presbyopia‐correcting cataract surgery, it is essential for the surgeon to evaluate the patient's personality, understand their expectations, and identify their specific needs for near vision in daily activities and hobbies. Following this comprehensive assessment, the surgeon can determine whether to aim for partial presbyopia correction, which enhances intermediate vision but may still require the occasional use of reading glasses for near tasks, or full presbyopia correction, which improves both intermediate and near vision and provides genuine independence from glasses. The surgeon should then select the most appropriate IOL type and surgical technique to achieve the desired visual outcomes.

Technologies that simulate postoperative vision aim to help establish realistic expectations by allowing patients to preview what their vision might be like after surgery. However, the effectiveness of these simulations can be limited by cataract opacity, which complicates preoperative assessments. Despite these limitations, new simulation devices, such as SimVis Gekko (2EyesVision SL, Madrid, Spain) and VirtIOL (10Lens S.L.U., Terrassa, Spain), are demonstrating promise [23] even when used with cataractous lenses [24]. Nevertheless, further research is necessary to evaluate the performance of these technologies thoroughly [25].

Reimbursement policies often favour multifocal spectacles over premium IOLs for managing presbyopia, classifying these lenses as luxury items accessible only to the wealthy. However, the perception of high costs associated with these ‘premium’ IOLs is frequently overstated. In reality, the expense of these lenses is often comparable to—or even lower than—that of an advanced smartphone, a device most people regularly purchase and replace every few years. Highlighting the long‐term benefits of premium IOLs is crucial, as they provide lifelong improvement in near vision at a reasonable price. As emerging evidence suggests that the lifetime societal costs of managing presbyopia with spectacles exceed the one‐time investment in premium IOLs [26], reimbursement policies may shift, making these lenses more widely accessible.

A comprehensive patient examination is critical before performing presbyopia‐correcting cataract surgery. The patient's baseline vision—shaped by cataract severity and refractive state—acts as a psychological benchmark for assessing postoperative improvement, a factor often overlooked by surgeons. Thorough clinical evaluation and detailed anamnesis are essential to rule out contraindications. Special attention should be paid to ocular surface dryness, as it can compromise the accuracy of preoperative measurements and diminish the effectiveness of presbyopia‐correcting IOLs; treating dryness before reassessment is often necessary. Assessing eye anatomy is also important; atypical anatomical features can lead to higher targeting errors, with pupil diameter and corneal spherical aberrations (SAs) playing significant roles. Angle kappa and angle alpha should be considered due to their impact on IOL alignment and visual outcomes. Effective astigmatism management is crucial, favouring predictable techniques like toric monofocal lenses over less reliable methods such as limbal relaxing incisions (LRIs).

In our review, we have chosen to categorise IOLs based on their ability to enhance depth of focus: monofocals, enhanced monofocals, extended depth of focus (EDOF) lenses and multifocals. Monofocal lenses can partially correct presbyopia through mini‐monovision or full monovision techniques. Enhanced monofocals extend depth of focus but do not meet the criteria to be classified as EDOF lenses [27]. EDOF lenses provide intermediate vision and partial depth of field, offering partial presbyopia correction, while multifocal lenses enable near vision correction and deliver a full depth of field [28] or full presbyopia correction. We do not discuss accommodative IOLs, as they are not considered a viable solution in current management practices.

Proper classification of presbyopia‐correcting IOLs is essential due to widespread confusion. A key issue is the lack of manufacturer transparency about optical properties and the impact of marketing, which often uses new terminology to present supposedly innovative technologies. Some manufacturers call their lenses EDOF to avoid the negative associations with halo effects in multifocal IOLs [29]. Regulatory inconsistencies add to the confusion, as lenses classified as enhanced monofocals in some regions may be labelled EDOF elsewhere. Some diffractive EDOF lenses are described as ‘low near‐add multifocals’, a term we discourage [30]. In addition, certain multifocal IOLs are ambiguously labelled as both EDOF and multifocal [31] to highlight continuity in depth of focus, but smooth depth of focus is also a feature of newer pentafocal diffractive IOLs, which remain classified as multifocal [32].

We include in our classification an optical terminology that describes the technology used to achieve static accommodation and split light into various focal points or ranges, whether through refractive or diffractive means. In contrast to Ribeiro et al. [28], we believe that a classification system incorporating both optical and functional aspects of IOLs is essential, as it emphasises that optical technology provides significant clinical insights. For instance, diffractive lenses are commonly associated with an increased incidence of halos, while refractive lenses generally demonstrate greater pupil dependence.

Our classification offers the advantage of specifically targeting clinical objectives without neglecting the scientific aspects, which significantly influence outcomes and necessitate proper understanding for effectively managing these techniques and technologies. As ophthalmologists, it is essential not only to adopt a clinically oriented classification system for IOLs but also to thoroughly understand and clearly communicate the underlying scientific principles, optical concepts and technological innovations associated with each lens type. Such dual expertise reflects the evolving role of ophthalmologists, who increasingly must integrate sophisticated optical technologies into clinical practice. Ophthalmologists must balance complex technological considerations with comprehensive clinical, biological, and psychological assessments. This integrated approach ensures that patients receive highly personalised and effective presbyopia‐correction solutions, precisely tailored to their individual visual requirements, expectations and lifestyles. In this review, we focus on the clinical aspects of various techniques and presbyopia‐correcting lenses, as well as recent advancements within each. For a comprehensive review of presbyopia‐correcting IOL models and designs, readers are referred to our previous review [29]. Table 1 summarises all the available options.

**TABLE 1:** Comparative overview of surgical techniques for presbyopia correction: Methods, benefits and limitations.

Presbyopia correction technique	Correction level	Description	Advantages	Disadvantages
Monovision (blended vision)	Partial	Myopia induced in the non‐dominant eye (mini or full monovision), possibly combined with extended depth‐of‐focus (EDOF) or light‐adjustable lenses (LAL)	Established, simple, cost‐effective [33, 34]	Reduced stereopsis/depth perception [35, 36, 37, 38] Anisometropia causing visual discomfort, halos, headaches, possible strabismus aggravation [39, 40] Requires precise refractive outcome and astigmatism correction in dominant eye [41]
Enhanced monofocal lenses	Partial	Subtle variation of refractive power across the lens surface to extend slightly depth‐of‐focus	Good distance vision with improved intermediate vision Low risk, few contraindications	Limited near vision improvement Minimal perceived benefit compared to monofocal lenses [42] High cost relative to minor improvement
Refractive EDOF lenses	Partial	Variation of refractive power across the lens surface to extend depth‐of‐focus	Fewer dysphotopsia compared to multifocal intraocular lenses (IOLs) Effective intermediate vision, maintains stereopsis	Highly pupil‐dependent performance Susceptible to spherical aberration effects (especially with large pupils) Possible myopic shifts dependent on lighting conditions
Diffractive EDOF lenses	Partial	Elongation of focal range using diffractive gratings on the IOL surface	Less affected by pupil size and corneal spherical aberrations	More dysphotopsia compared to refractive EDOF Less visual gain compared to multifocals; patient tolerance lower due to modest benefit
Small‐aperture lenses (pinhole IOLs)	Partial	Reduce pupil aperture to increase depth‐of‐focus (pinhole effect), enhancing sharpness by filtering peripheral optical aberrations	Less impacted by corneal astigmatism and high order aberrations Suitable for irregular corneas	Reduced retinal illumination Impaired night vision Contraindicated in retinal pathology
Diffractive multifocal IOLs	Full	Diffractive optics creating distinct focal points (typically trifocal: near, intermediate, far)	High spectacle independence Good intermediate and near vision	Significant halos, glare, starbursts (particularly in low‐light conditions) Slight contrast loss due to high diffractive orders [18] Sensitive to ametropia (myopia or astigmatism) [43, 44] Generally contraindicated with ocular surface disorders, irregular astigmatism, or retinal pathology
Rotationally symmetric refractive multifocal IOLs	Full	Symmetrical refractive optics creating multiple focal points (bifocal/trifocal)	Reduced halos/glare compared to diffractive multifocal lenses Promising newer prototypes less pupil dependent [45]	Insufficient intermediate vision in older bifocal designs
Rotationally asymmetric refractive multifocal IOLs (including spiral IOLs)	Full	Asymmetrical refractive optics, including segmented refractive and spiral designs	Reduced halos/glare compared to diffractive multifocals Spiralisation allows an additional focal point	Image distortions, ghost images, coma [46] Spiral IOLs are newer concepts under evaluation
Complementary multifocal IOLs	Full	Paired multifocal lenses optimised differently in each eye (enhanced intermediate vs. enhanced near vision)	Tailored bilateral solution for extended binocular DOF Potentially enhanced intermediate and near vision	Possible reduction in distance visual acuity [47] Uncertain effect on binocular summation Limited evidence for superiority over identical bilateral implants [30]
Mix‐and‐match approaches	Full	Combination of different IOL types (monofocal + EDOF or multifocal lenses) also known as ‘hybrid monovision’, or two different types of presbyopia‐correcting IOL also called ‘premium’ monovision [48, 49]	Maximised visual benefits by leveraging strengths of different lens technologies Individualised visual optimisation	Potential compromised stereopsis at certain distances [36] Requires precise patient selection and careful planning [48, 50] Limited robust clinical evidence
Sulcus‐fixated supplementary IOLs (duet procedure)	Full	Secondary multifocal lens implanted in sulcus anterior to primary monofocal or toric lens [51]	Reversible multifocality (removable if unsatisfactory) [51, 52, 53] Option post‐monofocal cataract surgery for multifocal upgrade without full IOL exchange	Increased surgical complexity Risk of pigment dispersion, iris damage, interlenticular opacification Increased number of refractive surfaces causing additional light loss through reflection

### 3.1 Partial Presbyopia Correction

#### 3.1.1 Monovision Approach

Monovision, also called blended vision, the earliest technique for presbyopia correction in lens replacement surgery, was first studied and documented in 1984 [33]. This approach involves inducing myopia in one eye and has been implemented through various methods, including glasses, contact lenses, corneal refractive surgery and monofocal IOLs.

Myopia is typically induced in the non‐dominant eye, as determined preoperatively. It is important to note, however, that ocular dominance may change following cataract extraction. As cataracts progress, patients often adapt to visual changes over time, which can lead to a shift in ocular dominance, particularly when the cataract is asymmetrically more severe in the dominant eye. Postoperative shifts in ocular dominance occur in approximately 20%–40% of cases [54, 55], potentially resulting in crossed monovision, where the non‐dominant eye assumes the role of managing distance vision. Nevertheless, the overall impact of crossed monovision does not appear to significantly affect overall visual outcomes [56, 57, 58].

Despite remaining a widely used option among surgeons [34], monovision has notable disadvantages that are often underestimated. A primary drawback is the reduction in stereopsis, or depth perception, between the eyes [35], which negatively impacts overall visual quality. Achieving good visual acuity alone is insufficient; adequate stereopsis is crucial [36], especially for older adults, as impaired depth perception is a recognised risk factor for falls [37] and hip fracture [38].

In addition, anisometropia induced by monovision disrupts binocular summation—the brain's process of merging images from both eyes to create an improved single visual perception. While a perfectly emmetropic eye may provide sufficient distance visual acuity, even with a monovision correction of −2.5 D, reductions in contrast sensitivity and patient‐reported visual quality are noted with refractive differences as small as −0.75 D [59]. This involves carefully selecting patients to minimise the risk of refractive discrepancies, using advanced formulae, and conducting comprehensive preoperative evaluations. The use of light‐adjustable lenses (LALs) can be beneficial in achieving precise emmetropia [60]. Any degree of astigmatism, even small, should be corrected in the dominant eye [41]. However, slight refractive errors in the nondominant eye are less critical, and low astigmatism, particularly against‐the‐rule (ATR) astigmatism, has been shown to improve near vision [61, 62] and may be acceptable.

Another important point to consider is that monovision requires the brain to alternate between the two eyes for near and distance vision. In patients with a history of strabismus or significant phoria, this adjustment may lead to decompensation years after surgery due to the refractive error discrepancy between the two eyes. Such a situation can result in patient dissatisfaction and the development of new‐onset strabismus, which might be correctable with glasses that negate the effects of monovision but could also necessitate strabismus surgery [39]. This rare complication may be underreported, as it can manifest several years postoperatively.

To address these issues, the degree of anisometropia can be reduced using mini‐monovision, which induces a smaller amount of myopia, typically ranging from −0.75 to −1.25 D. This approach helps to preserve better stereopsis [63] but is associated with a lower rate of spectacle independence [64]. Mini‐monovision can also be combined with EDOF lenses to enhance depth of focus [65]. However, the outcomes are mixed: some studies report no significant disadvantages [66], while others note potential drawbacks, such as reduced distance vision and the presence of additional halos caused by low myopia in the contralateral eye [40].

#### 3.1.2 Enhanced Monofocal Lenses

Enhanced monofocal IOLs are gaining popularity for bilateral implantation, owing to their potential advantages over traditional monofocal IOLs. These lenses provide excellent distance vision with improved intermediate acuity; however, their performance does not match that of trifocal or EDOF lenses [67]. In addition, the improvement over standard monofocal IOLs may be subtle enough to go unnoticed [42]. Surgeons appreciate these lenses because they feel they can address presbyopia with minimal risk to the patient and relatively few contraindications.

#### 3.1.3 Extended Depth‐of‐Focus Lenses

##### 3.1.3.1 Refractive EDOF Lenses

Most contemporary refractive EDOF IOLs extend the range of vision by incorporating higher optical power in the central zone of the lens. This central zone can be designed as a small central ‘pastille’, an annulus, or more complex patterns. The modification of central power effectively increases the depth of focus by directing central rays and adjusting them for near vision. Similarly, EDOF lenses that enhance negative SA utilise the same fundamental mechanism, though with smoother surface profiles. It is worth noting that all refractive EDOF lenses shape the wavefront, and the term ‘wavefront shaping’ is often employed as a marketing label rather than being grounded in robust scientific evidence.

**FIGURE 1:**
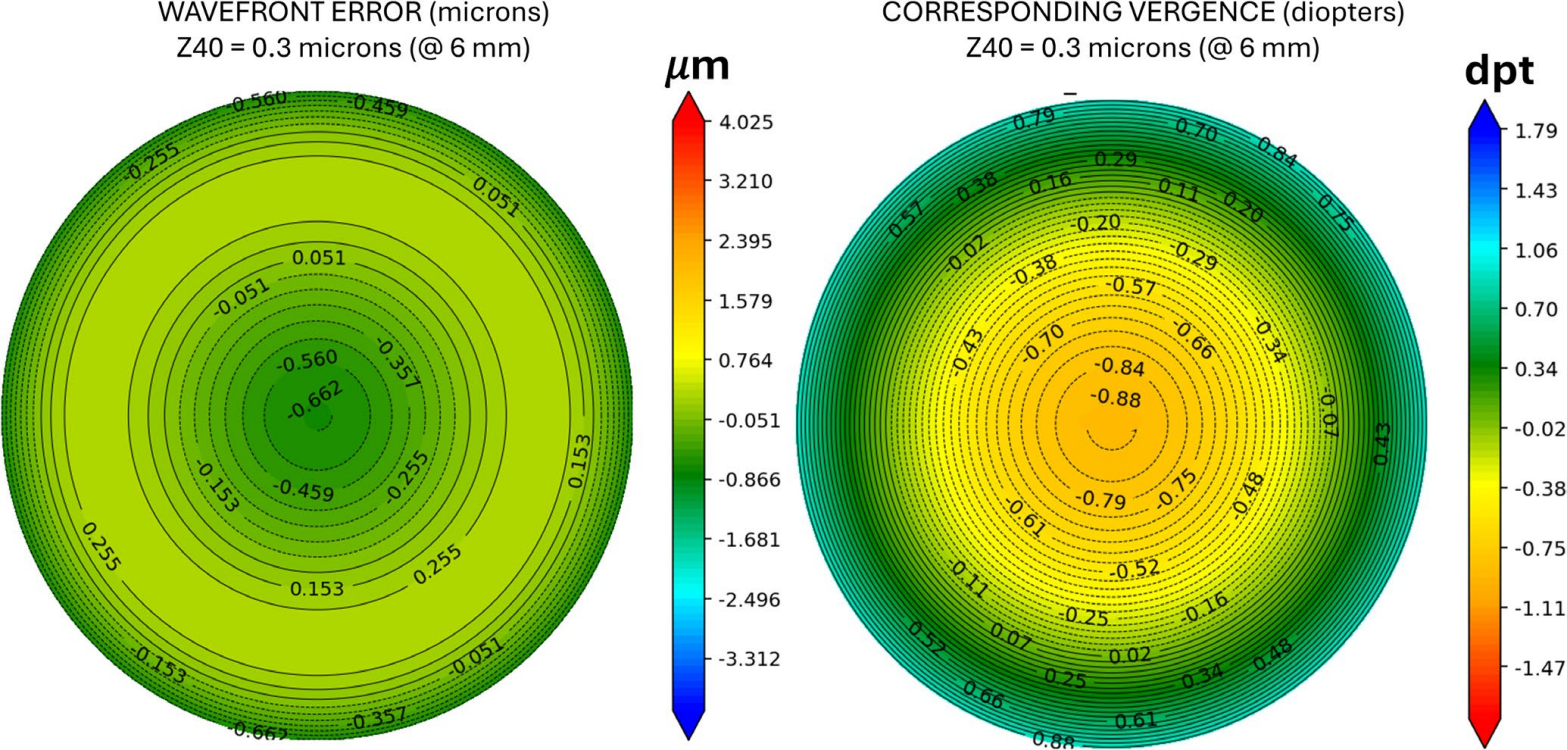
Wavefront and vergence representation for −0.3 μm Zernike spherical aberration (SA) for a 6 mm pupil diameter. The left map shows the wavefront of a −0.3 μm Zernike SA, exhibiting a sombrero‐like shape with central depression and peripheral elevation. While this micron‐scale representation lacks direct refractive interpretation, the right vergence map (in diopters) illustrates the refractive effect: Central myopisation (−0.88 D) transitioning to peripheral hyperopisation (+0.88 D). This negative SA results in a progressive optical power reduction from centre to periphery, corresponding to a ~2 D decrease across the pupil. This gradual shift enhances depth of field while minimising defocus for distance vision.

A significant drawback of refractive EDOF lenses is their dependence on pupil size and the interaction of IOL SA with their performance. Indeed, after implantation of an EDOF lens inducing negative SA, patients with small photopic pupil sizes may experience a myopic refractive surprise or daytime myopia, with myopia occurring only under photopic conditions. In patients with large pupils, the effect of the central element can be reduced and patients may complain about poor intermediate vision. Figure 1 illustrates the refractive impact of a −0.3 μm negative SA in a 6 mm pupil, as a function of ray position within the pupil.

Moreover, in patients with large pupils, corneal SA can have a significant impact: high positive corneal SA can counteract the increased depth of focus provided by these IOLs, whereas low corneal SA (or negative SA) can enhance the depth of focus but reduce visual quality.

Pupil dependence arises because the refractive power varies according to the radius and concentric zones of the lens. While the addition of power undulations through extra concentric zones can mitigate this pupil dependence, emerging designs such as light sword lenses use angular modulation of optical power [68]. These innovative designs offer the prospect of pupil independence and may become a promising solution for presbyopia correction [69].

Moreover, while the EDOF effect is currently achieved by modifying the lens shape, future advancements may involve gradient index (GRIN) lenses [70]. These lenses generate the EDOF effect through variations in the refractive index within the lens material. Although technically challenging, GRIN lenses hold considerable promise. This potential is underscored by LALs, which, after polymerisation, create zones with different refractive indices that induce negative SA and produce an EDOF effect [60].

##### 3.1.3.2 Diffractive EDOF Lenses

Diffractive EDOF lenses feature kinoform gratings that cause diffraction, but they have a larger step width (hence fewer steps), causing the light to diffract to a focus with less power than full range diffractive multifocal lenses. Diffractive EDOF implants are specifically designed with higher diffractive steps to address chromatic aberration caused by refractive elements. This approach leverages the fundamental difference between the chromatic effects of refraction and diffraction. In refractive optics, chromatic aberration occurs because shorter wavelengths (blue light) are bent more strongly than longer wavelengths (red light), leading to a focal shift that can degrade image quality. Conversely, diffraction has the opposite effect: shorter wavelengths are diffracted less than longer wavelengths.

**FIGURE 2:**
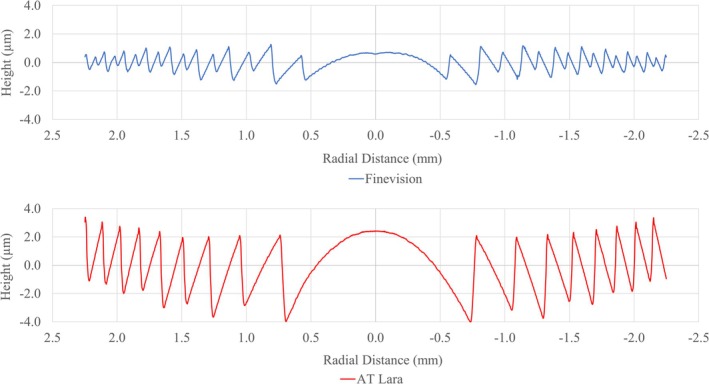
Diffractive step profiles provided by the NIMO TEMPO (Lambda‐X, Nivelles, Belgium) from the wavefront measurements of the multifocal lens FineVision HP (BVI) and the diffractive EDOF AT LARA 829 (Zeiss). The profile shows that the AT LARA has wider steps, resulting in a lower addition to the lens. However, the steps are also higher, which enhances light diffraction and intends to correct for chromatic aberration.

By carefully designing the height of the diffractive steps, the chromatic dispersion introduced by diffraction can be tuned to counteract the chromatic aberration produced by refraction. This compensatory mechanism helps to minimise the overall chromatic aberration in the visual system (Figure 2) However, this design may pose challenges for patients, as the lack of near‐vision benefits typically offered by multifocal lenses can make halos induced by EDOF lenses more difficult to accept.

##### 3.1.3.3 Small‐Aperture Lenses

Small‐aperture lenses, also known as pinhole IOLs, are designed to reduce the effective aperture size created by the pupil. By decreasing pupil diameter, these lenses filter out peripheral light rays, which are more susceptible to optical aberrations, thereby allowing only central rays to reach the retina. This selective filtering enhances image sharpness via reduction of the blur patch and reduces susceptibility to defocus, augmenting depth of field by minimising optical distortions.

This effect is described in optical systems using the *f* number, which is calculated as the focal length divided by the aperture diameter. A reduction in aperture size leads to an increased *f* number. Although this principle is beneficial in photography—where longer exposure times can offset reduced light intake—in the human eye, it results in decreased light entry. This reduction may impair visual acuity under low‐light conditions, posing a significant challenge in the application of small‐aperture lenses for vision correction.

### 3.2 Full Presbyopia Correction

Full correction of presbyopia can be achieved through bilateral implantation of multifocal IOLs or through a mix‐and‐match strategy, which involves unilateral implantation of a multifocal IOL combined with a different contralateral IOL. Another approach is using monovision with EDOF lenses.

Multifocal IOLs have seen substantial advancements, evolving from early refractive technology—similar to that utilised in multifocal contact lenses—to predominantly diffractive technology in most modern designs. Furthermore, these lenses have progressed from bifocal to trifocal configurations, enhancing intermediate vision without sacrificing distance or near vision [71].

The successful implantation of multifocal IOLs requires precise refractive outcomes, achieved through accurate power calculations and meticulous management of astigmatism. Utilising a myopic target as a margin of error is not recommended for these lenses, as even a slight myopic refractive error can detrimentally affect their optical performance. Instead, aiming for an emmetropic or even slightly hyperopic target is recommended to optimise visual outcomes [43]. Moreover, multifocal IOLs are notably more sensitive to astigmatism compared to EDOF lenses [44], with even minimal astigmatism substantially compromising optical performance [72].

#### 3.2.1 Diffractive Multifocal Lenses

Diffraction technology offers the advantage of reduced dependence on pupil size. Notably, it can even leverage controlled pupil dependence through apodisation, as demonstrated in the FineVision HP, BVI (Figure 2), where the step height is progressively reduced towards the periphery. This modulation reduces near vision under mesopic conditions while maintaining high‐quality distance vision, which is critical for tasks such as night driving.

However, multiple diffraction orders can cause light dispersion, slight image contrast loss, and may induce hazy vision, as previously reported by wearers of discontinued diffractive contact lenses. Nonetheless, this effect is less critical for diffractive multifocal IOLs, likely because cataract patients, having had their cataractous lenses removed, are less sensitive to these contrast losses since they experience overall vision quality improvement [18].

#### 3.2.2 Rotationally Symmetric Refractive Multifocal Lenses

Rotationally symmetrical refractive bifocal lenses have been less commonly used, but these designs may see a resurgence with the development of new prototypes, such as periodic refractive EDOF lenses. These designs feature multiple concentric rings and radial power undulations, offering up to 2.25 D of extended depth of field, placing them in the multifocal IOL category. From a clinical perspective, this brings them closer to multifocal lenses rather than traditional EDOF lenses. These new designs demonstrate notable advantages, including reduced pupil dependency compared to classic refractive EDOF lenses and fewer halos compared to diffractive designs [45].

#### 3.2.3 Rotationally Asymmetric Refractive Multifocal Lenses

Traditionally, rotationally asymmetric refractive multifocal IOLs were limited to bifocal sectoral designs featuring an additional power segment. However, recent advancements in IOL technology have renewed interest in this category of multifocal lenses, particularly with the introduction of the recently first commercialised spiral IOL.

Bifocal sectoral designs present unique optical challenges, including a distinct dysphotopsia profile and potential image smear. Patients frequently report experiencing starbursts and ghost images, while halos are described less often. These ghost images—sometimes referred to as decoupling—consist of an additional image, particularly in the visual field corresponding to the supplementary segment, where peripheral defocus is more pronounced [46]. To mitigate these effects, manufacturers typically recommend inferonasal implantation. This recommendation is theoretically justified, given that the inferonasal field is naturally restricted by nasal anatomy. However, clinical studies have shown no significant difference between inferonasal and superotemporal placement [73]. In fact, positioning the lens superotemporally in the dominant eye may offer advantages, as it distances the near segment from the visual axis and potentially leverages the natural angle alpha [74].

In terms of image distortion, these designs have been measured to induce significant odd higher‐order aberrations such as coma, as demonstrated by postoperative wavefront measurements [75]. Nonetheless, recent evidence suggests that this observed coma may partly result from measurement bias and inaccuracies in wavefront analysis, which do not account for the abrupt power transitions characteristic of bifocal designs [76]. Nevertheless, asymmetric designs and these abrupt power transitions can still cause image distortion, which the brain does not always adequately correct when processing visual information.

Spiral IOLs utilise the principle of spiralisation, involving at least two refractive structures of different powers to create a ‘vortex effect’ through the peripheral wrapping of light rays, thereby producing an extended focal zone. The concept of spiral optics, initially described by Jones and Clutterbuck [77] and later fully formalised by Galinier et al. [78], utilises refractive rather than diffractive principles and is believed to have the advantage of being less sensitive to pupil size compared to conventional refractive multifocals [78]. This principle was incorporated into the first commercially available spiral IOL, which was further modified to include a central zone specifically designed to enhance distance vision.

#### 3.2.4 Complementary Multifocal IOLs

Complementary multifocal IOLs differ from mix‐and‐match strategies in that they consist of pairs of IOLs specifically designed for bilateral implantation in the same patient. This approach is currently employed using trifocal diffractive technology. By maintaining the overall lens geometry and minimising the modifications to the diffractive trifocal design, manufacturers can finely tune the optical behaviour of each lens—one optimised for intermediate vision and the other for near vision. These modifications may include adjusting the height of the diffractive gratings or altering the spacing of certain gratings to direct more light to a specific focal point.

This advanced technique may allow patients to achieve good distance vision in both eyes, with distance vision as the primary refractive focus of the two lenses, while one eye is optimised for intermediate vision and the other for near vision. Through binocular integration, the brain can theoretically merge these visual inputs, resulting in enhanced binocular vision with an extended and functional depth of field. The use of trifocal lenses is believed to preserve binocularity; however, the impact of the relative weakness of one eye's focus on the other remains not fully understood. As seen with the blended monofocal strategy, such disparities may affect binocular summation and potentially lead to a reduction in binocular visual acuity.

The use of complementary multifocal IOLs has shown mixed outcomes and requires further investigation. Although some results are promising [79], implanting a multifocal IOL with higher additional power in the contralateral eye may decrease distance and intermediate visual acuity [47] and complementary IOLs may not be superior to conventional bilateral implantation [30]. Moreover, the impact of ocular dominance on these systems remains to be fully explored.

### 3.3 Mix‐and‐Match Approaches

Mix‐and‐match IOL implantation involves combining different lens types to optimise visual outcomes and address the limitations associated with each IOL. Implanting a monofocal IOL in the dominant eye and a presbyopia‐correcting IOL in the non‐dominant eye is often referred to as ‘hybrid monovision’ [48, 50]. In addition, using premium IOLs with distinct optical properties in each eye is sometimes termed ‘premium monovision’ [48, 49].

The variety of IOL types and their potential combinations is extensive, making objective comparisons challenging. These combinations include pairing an EDOF lens with a multifocal lens [49], using two refractive multifocal IOLs with different add powers [80], combining an enhanced monofocal lens with a multifocal lens [81] or using small‐aperture IOLs in conjunction with segmental refractive bifocal lenses [82], among other strategies. Comparisons between mix‐and‐match approaches and the conventional implantation of identical IOLs yield mixed outcomes, with some studies reporting improved visual acuity, while others note a decrease. Furthermore, stereopsis may be compromised at certain distances [36].

### 3.4 Sulcus‐Fixated Supplementary IOLs

Originally referred to as ‘piggyback’, the implantation of a secondary IOL over a monofocal IOL is now preferably termed polypseudophakia, reflecting significant advancements from the initial technique, with supplementary IOLs specifically designed for placement in the sulcus [83]. The original approach involved implanting both lenses within the capsular bag, which was associated with complications such as unintended hyperopic shifts and interlenticular opacification, particularly when acrylic lenses were used.

Sulcus‐fixated supplementary IOLs have a diameter ranging from 13 to 14 mm, providing enhanced stability within the sulcus. The majority of these lenses are composed of hydrophilic materials and are designed with angulated, rounded‐edge haptics to minimise the risks of complications, including iris damage, pigment dispersion and secondary glaucoma. Furthermore, certain models feature larger optic diameters, extending up to 6.5 mm, to prevent optic capture and reduce the risk of pupillary block.

Sulcus‐fixated supplementary IOLs are typically 13–14 mm in diameter, enhancing stability within the sulcus. Most of these lenses are hydrophilic and feature angulated, rounded‐edge haptics, which minimise risks such as iris damage, pigment dispersion, and secondary glaucoma. In addition, some models have larger optic diameters, up to 6.5 mm, to prevent optic capture and reduce the risk of pupillary block.

The ‘duet procedure’ is the simultaneous implantation of a multifocal supplementary IOL in the sulcus alongside a monofocal IOL in a single‐step approach. This technique offers a significant advantage: reversible multifocality [51, 52, 53]. It allows patients to trial multifocal technology with the option to remove only the supplementary IOL if they experience dissatisfaction, such as photic phenomena, without the need for a more invasive IOL exchange surgery. Furthermore, implantation over a toric IOL has been demonstrated to be both feasible and safe, without causing long‐term rotational instability [51].

The duet procedure is particularly advantageous for patients with moderate myopia who have high visual expectations for near tasks, as they are accustomed to excellent near vision. For these individuals, a myopic target can be selected for the monofocal implant, which is then compensated by a negative‐power supplementary IOL. If the multifocal supplementary IOL is later removed, the patient's myopic correction is preserved, ensuring continued satisfactory near vision [51, 53].

Furthermore, for patients who have previously undergone cataract surgery with a monofocal IOL and are dissatisfied with their near vision but are interested in multifocal technology, the implantation of a multifocal supplementary IOL as a secondary procedure provides a less invasive alternative, obviating the need for lens exchange.

## 4 Presbyopia Correction in Complex Eyes

Presbyopia correction in eyes with unusual anatomy or ocular diseases poses significant challenges. Monovision correction in cases with associated conditions such as corneal diseases, extreme axial lengths or prior corneal refractive surgery—is particularly complex due to the increased prevalence of refractive errors in these patients. The relative dissociation between eyes can negatively affect binocular summation, reduce tolerance to refractive error and compromise patient satisfaction, especially for distance vision. Postoperative corneal refractive enhancement may be required to achieve emmetropia in these cases [84]. While LALs represent a viable option for such patients, their availability remains limited for many surgeons [85].

Presbyopia‐correcting IOLs should be employed with caution in patients with ocular diseases or a history of eye surgery. The static accommodation provided by current presbyopia‐correcting IOLs represents an optical compromise, as these lenses superimpose images focused at varying distances, potentially reducing overall visual quality and contrast sensitivity. In addition, photic phenomena, a common postoperative occurrence with these lenses, may be exacerbated in certain conditions.

Several illustrative clinical scenarios underscore these considerations:

### 4.1 Case Study 1

A 70‐year‐old patient with stable age‐related macular degeneration (AMD) underwent polypseudophakic implantation, consisting of an in‐the‐bag monofocal IOL combined with a sulcus‐fixated supplementary multifocal lens. Postoperatively, the patient achieved satisfactory functional vision at far, near and intermediate distances. Unfortunately, the AMD progressed to the wet form after surgery. The sulcus‐fixated multifocal lens was subsequently removed through a straightforward surgical procedure, resulting in the restoration of optimal contrast sensitivity and visual function.

Polypseudophakia, which enables reversible multifocality, may provide a safer alternative by broadening the indications for multifocal IOLs while reducing the risks associated with IOL exchange, which can have a potentially devastating impact on an already diseased eye. This is particularly relevant in progressive diseases—such as AMD and epiretinal membrane (ERM)—where presbyopia‐correcting lenses are relatively contraindicated due to the unpredictable progression of these conditions.

### 4.2 Case Study 2

A 68‐year‐old patient with moderate Fuchs' dystrophy underwent cataract surgery with implantation of a trifocal presbyopia‐correcting IOL, despite a low endothelial cell count (1200 cells/mm^2^). Initially satisfied, the patient's vision deteriorated after 1 year due to corneal oedema. Subsequent Descemet Membrane Endothelial Keratoplasty (DMEK) surgery restored corneal clarity; however, a residual refractive error developed, leading to patient dissatisfaction. Post‐DMEK pachymetry was insufficient to permit laser vision correction, leaving glasses as the only viable solution.

The use of presbyopia‐correcting IOLs in patients with Fuchs' endothelial dystrophy remains controversial. Some experts recommend preoperative endothelial cell count assessment and advise against presbyopia‐correcting IOL implantation in this population [86]. Others suggest that these lenses can be implanted, even in patients requiring DMEK, preferring a staged approach. This involves initially performing DMEK to restore endothelial health, followed by a secondary implantation of the presbyopia‐correcting IOL to minimise postoperative refractive error postoperatively [87]. However, changes in DMEK function over time and the potential need for subsequent keratoplasty present significant challenges in these cases.

### 4.3 Case Study 3

A 67‐year‐old patient presented for cataract surgery with a history of LASIK performed several years previously at another centre. Given the patient's high visual expectations and prior spectacle independence, implantation of an EDOF IOL was initially considered. However, preoperative assessment identified significant negative SA attributable to prior presbyopic LASIK. Recognising that existing corneal multifocality already provided an inherent extended depth‐of‐focus effect, a monofocal IOL with neutral SA was selected and implanted. Postoperatively, the patient achieved excellent visual outcomes, including good distance and intermediate vision, minimal photic disturbances, and high satisfaction.

This case emphasises the importance of thorough preoperative evaluation in patients with a history of corneal refractive surgery to optimise surgical planning and outcomes. Patients with a history of presbyopic LASIK may face challenges when evaluating the implantation of presbyopia‐correcting IOLs. The corneal multifocality induced by presbyopic LASIK can reduce the need for presbyopia‐correcting lenses while potentially interfering with the performance of presbyopia‐correcting IOLs, which may compromise visual quality. Therefore, standard monofocal lenses may offer a more appropriate alternative [88]. The growing popularity of corneal refractive surgery among younger populations has resulted in an increasing number of patients presenting with a history of prior refractive procedures. Managing these individuals presents unique challenges due to their heightened visual expectations, as they have already undergone surgery to enhance their vision.

Refractive EDOF IOLs are employed by some surgeons based on the rationale that their broader landing zone and greater tolerance for residual refractive errors can address refractive uncertainty in these patients. However, higher‐order aberrations—most notably SA—resulting from prior refractive surgeries may compromise the optical performance and depth‐of‐focus functionality of refractive EDOF lenses. In certain cases, these lenses may even function comparably to monofocal lenses in such patients. To optimise outcomes, careful consideration should be given to the patient's previous laser treatment, corneal asphericity and pupil size.

EDOF and multifocal IOLs utilising diffractive technology are sometimes used; however, these lenses may theoretically exacerbate photic phenomena, such as glare and halos, which are often already present in these patients. Following the implantation of these lenses, photic phenomena have been reported in up to 60% of cases [89].

Another common scenario encountered in clinical practice involves macular diseases. Clinical evidence demonstrating contrast sensitivity loss after the implantation of multifocal IOLs remains limited [90]. Moreover, the retina's ability to adapt to contrast reduction appears to remain unaffected even in the presence of mild to moderate macular disease, suggesting that adaptation is feasible following the implantation of presbyopia‐correcting lenses [91]. However, since static accommodation theoretically reduces contrast by superimposing images at different focal distances, caution is warranted.

Small‐aperture IOLs, which reduce illumination, are widely recognised as contraindicated in any condition affecting retinal sensitivity. In contrast, other EDOF lenses are sometimes preferred by surgeons over multifocal lenses due to their perceived ability to minimise contrast sensitivity loss. Enhanced monofocal lenses, designed to offer modest improvements in intermediate vision, have also shown favourable outcomes, even in patients with ERM who have undergone vitrectomy [92].

## 5 Conclusion

Presbyopia correction has become a cornerstone of lens replacement surgery, driven by the increasing demand from patients to reduce or eliminate their reliance on reading glasses. In RLE procedures, addressing presbyopia is often not just a goal but the primary objective. Optimal surgical outcomes require a thorough understanding of each patient's unique needs, enabling a collaborative decision on the most appropriate approach, whether that involves partial or full correction of presbyopia.

Partial correction options include monovision and EDOF lenses. While monovision has its limitations, which are often underestimated, the implantation of EDOF lenses demands meticulous evaluation of the specific constraints and trade‐offs inherent to each technology. Refractive EDOF lenses can be influenced by pupil size and may interact unfavourably with corneal SA. Small‐aperture lenses can diminish light transmission and affect overall visual quality. Diffractive EDOF lenses may introduce halos that impact night vision and overall patient satisfaction.

For patients seeking full presbyopia correction, diffractive trifocal lenses offer satisfactory vision across a full range of distances. Emerging advancements in new refractive multifocal lens design may offer the potential for further improvements. In addition, mix‐and‐match strategies, which combine different types of lenses to capitalise on their respective strengths, show promise. However, further clinical research is needed to establish the safety and efficacy of these approaches.

In complex cases, such as those involving patients with diseased eyes or a history of corneal refractive surgery, presbyopia correction poses additional challenges. Long‐term strategies must be carefully considered, particularly for individuals with progressive ocular conditions. Polypseudophakia, with its potential for reversibility, may represent a compelling alternative approach.

The continually evolving field of presbyopia correction in lens replacement surgery highlights the importance of personalised care. Tailoring surgical strategies to the individual needs of patients is essential for achieving optimal results. As the number of available options increases, AI‐driven surgical planning may represent the next significant advancement. Advances in technology and ongoing clinical trials are crucial for refining techniques and expanding the range of effective solutions. By prioritising individualised treatment and keeping pace with innovations, surgeons can better address the diverse needs of their patients, ultimately improving visual function and quality of life.

## Conflicts of Interest

Damien Gatinel serves as a consultant for BVI Company. The other author declares no conflicts of interest.

## Data Availability

Data sharing not applicable to this article as no datasets were generated or analysed during the current study.
